# Exploring microproteins from various model organisms using the mip-mining database

**DOI:** 10.1186/s12864-023-09735-1

**Published:** 2023-11-02

**Authors:** Bowen Zhao, Jing Zhao, Muyao Wang, Yangfan Guo, Aamir Mehmood, Weibin Wang, Yi Xiong, Shenggan Luo, Dong-Qing Wei, Xin-Qing Zhao, Yanjing Wang

**Affiliations:** 1 State Key Laboratory of Microbial Metabolism, Joint International Research Laboratory of Metabolic & Developmental Sciences, School of Life Sciences and Biotechnology, Shanghai Jiao Tong University, Shanghai, 200240 China; 2 Central Laboratory of Yan’an Hospital Affiliated to Kunming Medical University, Kunming, 650051 China; 3 Shanghai Artificial Intelligence Laboratory, Shanghai, 200232 China; 4 Zhongjing Research and Industrialization Institute of Chinese Medicine, Zhongguancun Scientific Park, Meixi, Nayang, Henan 473006 China; 5 Peng Cheng Laboratory, Vanke Cloud City Phase I Building 8, Xili Street, Nanshan District, Shenzhen, 518055 Guangdong China; 6 Engineering Research Center of Cell & Therapeutic Antibody, School of Pharmacy, Shanghai Jiao Tong University, Shanghai, 200240 China

**Keywords:** Microprotein, RNA-Seq, Stress response, Disease, Mip-mining database

## Abstract

Microproteins, prevalent across all kingdoms of life, play a crucial role in cell physiology and human health. Although global gene transcription is widely explored and abundantly available, our understanding of microprotein functions using transcriptome data is still limited. To mitigate this problem, we present a database, Mip-mining (https://weilab.sjtu.edu.cn/mipmining/), underpinned by high-quality RNA-sequencing data exclusively aimed at analyzing microprotein functions. The Mip-mining hosts 336 sets of high-quality transcriptome data from 8626 samples and nine representative living organisms, including microorganisms, plants, animals, and humans, in our Mip-mining database. Our database specifically provides a focus on a range of diseases and environmental stress conditions, taking into account chemical, physical, biological, and diseases-related stresses. Comparatively, our platform enables customized analysis by inputting desired data sets with self-determined cutoff values. The practicality of Mip-mining is demonstrated by identifying essential microproteins in different species and revealing the importance of *ATP15* in the acetic acid stress tolerance of budding yeast. We believe that Mip-mining will facilitate a greater understanding and application of microproteins in biotechnology. Moreover, it will be beneficial for designing therapeutic strategies under various biological conditions.

**Supplementary Information:**

The online version contains supplementary material available at 10.1186/s12864-023-09735-1.

## Background

Microproteins, also called small proteins, or miniproteins, are encoded by small open reading frames (smORFs). Microproteins generally refer to proteins composed of up to 50 and 100 amino acids in prokaryotes and eukaryotes, respectively [1, 2]. Genes encoding such proteins are commonly presented in almost all domains of life, including bacteria, fungi, insects, plants, animals, and human microbiomes [2, 3]. However, related functional studies have been limited and even neglected, probably due to their small size and difficulty in detection due to low abundance and or special properties^3^. Recently, studies on microproteins as ‘dark matter’ in proteomics have received increasing attention [4]. Various studies have reported discovering and characterizing smORFs and microproteins in different living organisms, including microorganisms, plants, and humans [5–8]. It was has revealed that some microproteins are essential in cellular physiology, metabolism, development, cell signaling, and disease occurrence in various living organisms [9–15]. With the increasingly accumulated data available on the existence and expression of microproteins in multiple organisms, it will be feasible to unveil the functions and working mechanisms of this family of proteins.

Among the known functions of microproteins, cellular stress responses are of particular interest in various fields, including biology, biotechnology, and medical science [2]. Cells are confronted by constant changes in their external environmental conditions. During growth and metabolism, cells may encounter harsh environments, e.g., low pH, oxidative stress, high temperature, and toxins. Studies on microbial stress tolerance have received significant attention due to their implications in cell metabolism, environmental toxicity, food preservation, and fermentation efficiency to produce biofuels and biochemicals [16–20]. For example, the development of stress-tolerant yeast strains benefits efficient fuel ethanol production [21]. For higher eukaryotes such as plants and humans, failure to combat stressful environments leads to developmental deficiency and or diseases [18–20]. Therefore, stress tolerance has been an important topic for the developmental process, breeding crops, and disease treatment.

It has been reported that many microproteins participate in stress response and tolerance [2]. The development of efficient high-throughput gene manipulating methods, for example, CRISPR-based genome editing tools, has enabled rapid characterization of microprotein gene functions [11]. In addition, synthetic biology approaches can be employed to design and manipulate microproteins for improved phenotypes. Therefore, it can be expected that studying microprotein functions in stress response and tolerance substantially impacts microbial biotechnological applications, agriculture, longevity, and human health [22].

So far, there have been multiple databases collecting multi-layered information on microproteins, for example, the plant-related ones, namely, *Arabidopsis thaliana*-oriented microprotein database ARA-PEPs [23]; and plant-oriented microprotein database PsORF [24]; as well as SmProt [25, 26] which is based on eight model organisms (*Escherichia coli*, yeast, zebrafish, rat, mouse, fruit fly, *Caenorhabditis elegans*, and human) integrating multi-source microprotein data mainly in Ribosome profiling sequencing (Ribo-seq) data and mass spectrometry data. In addition, OpenProt [27, 28] was developed for small protein mining based on eukaryotes; TISdb [29] for alternative translation initiation in mammalian cells, and SORFs.org [30, 31], a database of small ORFs using Ribo-seq data. However, there are several limitations of the current databases: (1) The species covered by the databases mentioned above are limited in specific domains of life (mostly plants, microbes and or animals); (2) Most of these databases only provide search results and cannot perform personalized analysis [32–34]; (3) Transcriptomic data have been largely overlooked. Transcription regulation is critical for gene expression, and transcriptome data are abundantly available, which benefits exploring differential transcription of possible microprotein-encoding genes and their related genes for functional characterization [35]. (4) No database has been developed to explore microproteins involved in responses to environmental stress and diseases, which are critical to sustainable bioproduction and disease treatment.

To address the above limitations, we have developed a microprotein mining database called Mip-mining, and made a collection of 336 sets of RNA-seq data from species ranging from *Escherichia coli* to humans. The database presented here is designed explicitly for probing microprotein functions, which enables locating functional microproteins under stress conditions in a particular species or various diseases, especially cancers. Our database benefits the exploration of microprotein functions in stress response and disease occurrence, which are receiving increasing attention in various fields [36, 37]. We also demonstrate the identification of essential microproteins in budding yeast, plants, and humans using Mip-mining.

## Construction and content

### Database content

A total of 336 sets of data were deposited in the current version of our database covering nine species, including *A. thaliana*, *E. coli*, *Oryza sativa*, *Saccharomyces cerevisiae*, *C. elegans*, *Danio rerio*, *Drosophila melanogaster*, *Mus musculus*, and *Homo sapiens*. Each set of the data contains specific information: the GSE Accession of the RNA-seq data in the GEO database, the stress type of the experiment, the sample number of the data, and the source of the RNA-seq data, including the GSE title with the corresponding link. Each data set has been manually checked and processed using a high-performance computing platform through a standard RNA-seq analysis process. Redundant intermediate files are deleted to save the time of users and computer storage space.

### Data collection and organization

To reveal the relationship between microprotein and its function, we chose to collect stress-related data by using keywords such as “stress” and “response to” to search in the GEO database [38]. Human diseases such as “diabetes” and “cancer” are also related to stress [39], so we also added these data. The data as a whole has been manually checked to ensure that it retains the original data and that it belongs to RNA-seq files. Additionally, the corresponding literature was also checked to confirm whether the results were related to stress. For a dataset to be included in our database, the corresponding relevant dataset was selected to meet the following predefined inclusion criteria: (1) The original SRA file is available; (2) a related study for related research has been published and can be tracked; and (3) enough relevant RNA-seq data is available to construct at least one comparison model. Finally, we categorized the data according to species and stress types. The number of stress types in the database is listed in Table 1.

**Table 1:** Number of stress conditions per species

Species	Biological response	Chemical treatment	Disease	Multiple stresses	Physical stress
*S. cerevisiae*	4	37	0	3	6
*O. sativa*	2	3	0	0	14
*E. coli*	0	37	0	3	6
*A. thaliana*	0	6	0	0	22
*M. musculus*	3	29	0	0	6
*H. sapiens*	3	17	91	0	9
*D. rerio*	3	12	0	0	9
*C. elegans*	6	8	0	0	9
*D. melanogaster*	1	9	0	0	2

### Reference genome resources and reference microproteins

Each species’ reference genome and annotation files were downloaded from GENCODE [40], Ensembl [41], and the NCBI-Genome database. The downloaded files contain reference genome fasta data, index data during Hisat2 [42] alignment, and general feature format (gtf) data.

Reference microproteins were obtained by a two-step screening. First, all microproteins ($\le$100 AA) related to each species were downloaded from the UniProt database (https://www.uniprot.org/). Importantly, considering that most microproteins are large protein fragments or recognizable subunits, we performed a second round of screening and obtained high-confidence reference microproteins.

### Expression matrix retrieval

We used the standard RNA-seq procedure to process the selected high-quality transcriptomic data. Sratoolkit (https://github.com/ncbi/sra-tools/wiki) is a toolkit provided by NCBI for processing sequencing data from the SRA database (Sequence Read Archive database [43]), and we used its built-in plugins for data processing. The Prefetch (version 2.10.9) was used to download the data, and fasterq-dump (version 2.10.9) assisted in decompressing data. In terms of quality control, we used FastQC (version 0.11.9) (http://www.bioinformatics.babraham.ac.uk/projects/fastqc) to check data quality, multiQC (version 1.9) [44] to integrate data quality files, and fastp (version 0.19.5) [45] to cut low-quality fragments to ensure the quality of each set of information. Next, we aligned the sequencing files to the reference genome using Hisat2 (version 2.2.1) [42], and we employed StringTie (version 2.1.4) [46] to generate merged transcripts, before converting them to the format adapted for the downstream processing R package called ballgown (version 2.18.0) [47].

### Differential expression and enrichment

We conducted statistical analyses in the R environment (Version 3.6.1, http://cran.r-project.org/). Several R packages were used; for instance, the “ballgown” (version: 2.18.0) constructed the gene FPKM expression matrix; for principal component analysis, we used “factoextra”(version: 1.0.7) [48] and “FactoMineR” (version: 2.4) [49] packages for data dimensionality reduction. The differentially expressed microproteins were screened using the “limma” package (version: 3.42.0) [50]. Downstream enrichment analysis, including GO, KEGG, and GSEA annotations, are performed through these packages: “enrichplot” (version:1.6.0) [51], and “clusterProfiler” (version:3.14.0) [52]. Visualization of analysis results is achieved by integrating the “ggplot2” package (version: 3.3.3) [53] with the “ggrepel” package (version:0.9.1) [54].

### Back and front-end design

**Fig. 1:**
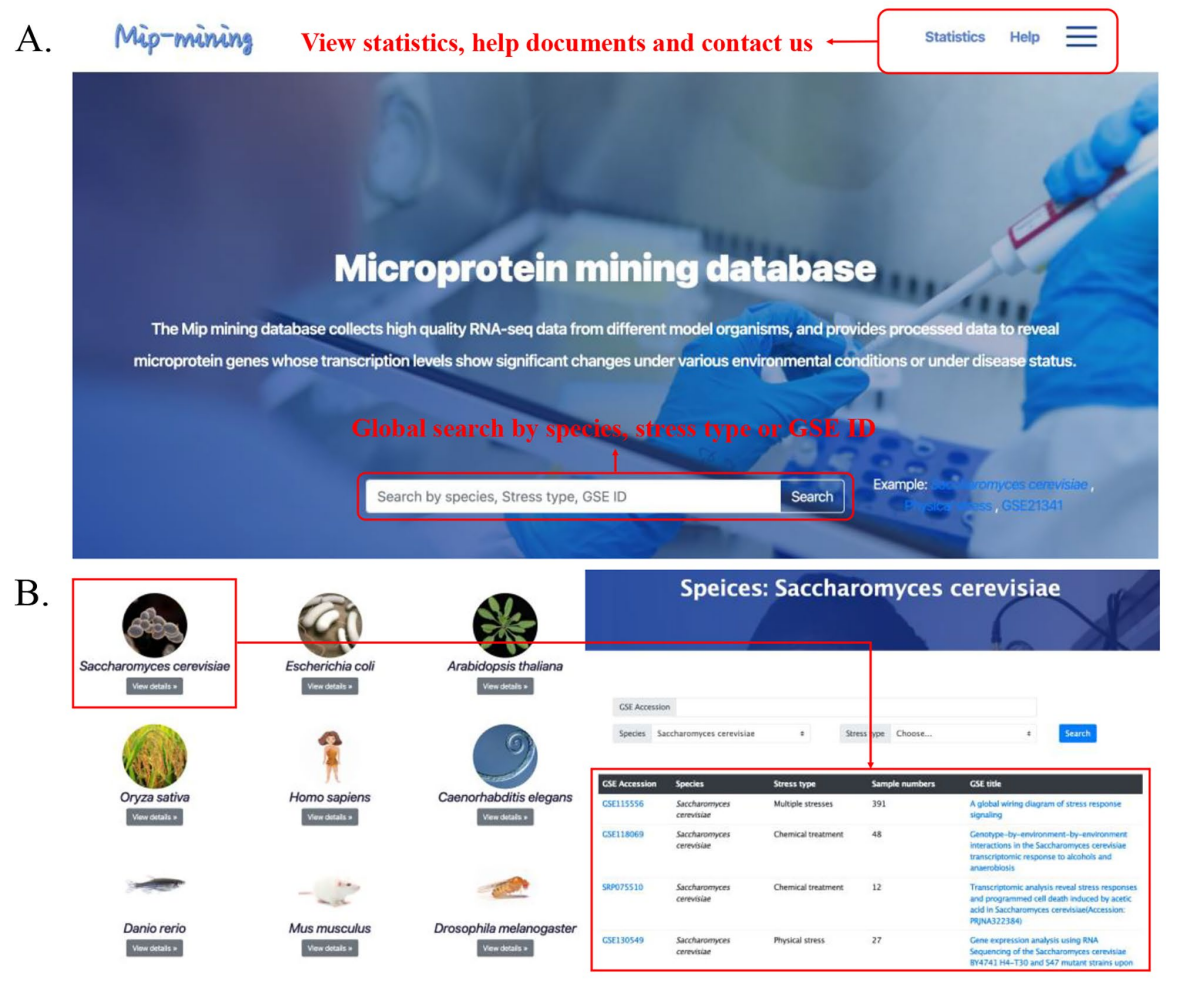
User interface of the Mip-mining database. (**A**) Global search function and related information are provided on the home page. Mip-mining offers a platform to search by species, stress type, and GSE ID. (**B**) Browse specific species data from Mip-mining

**Fig. 2:**
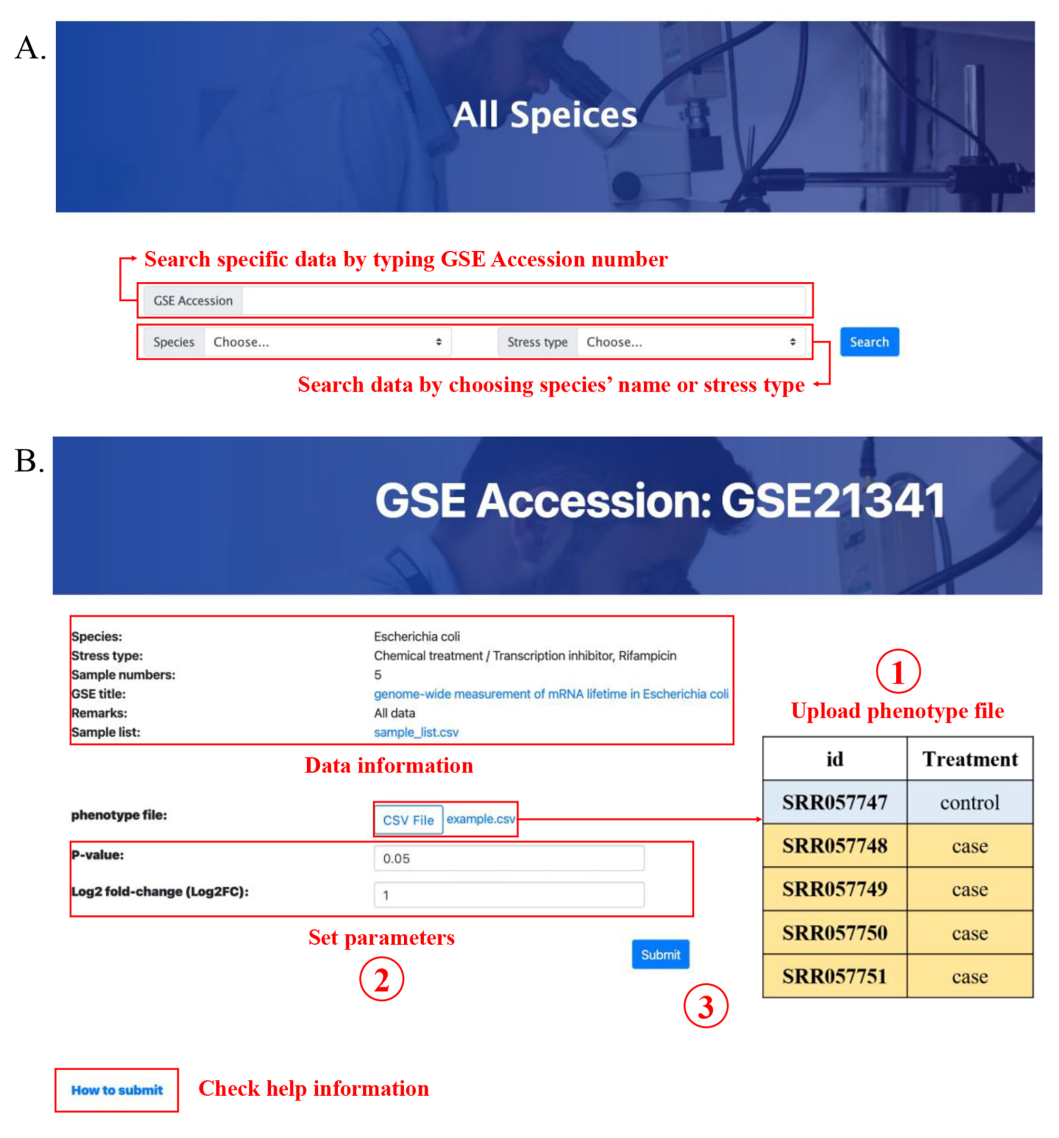
User interface of the Mip-mining database. (**A**) Select search methods on the all-species page. (**B**) Personalized analysis on the analysis page. This analysis needs uploading the corresponding phenotype file, setting analyzed parameters, and then clicking the submit button. Users can also click “How to submit” to check help information

The Microproteins mining database provides a user-friendly web interface that enables users to search and retrieve microprotein-stress function associations in the database (Fig. 1. and Fig. 2.). All data in the Microproteins mining database were stored and managed using MySQL (version 5.5). The web interfaces and services were built using Tomcat 8, JDK 1.8, and Bootstrap 3. Some exemplary use cases showing the utility of Mip-mining are available at https://weilab.sjtu.edu.cn/mipmining/help.

## Utility and discussion

### Architecture of Mip-mining

**Fig. 3:**
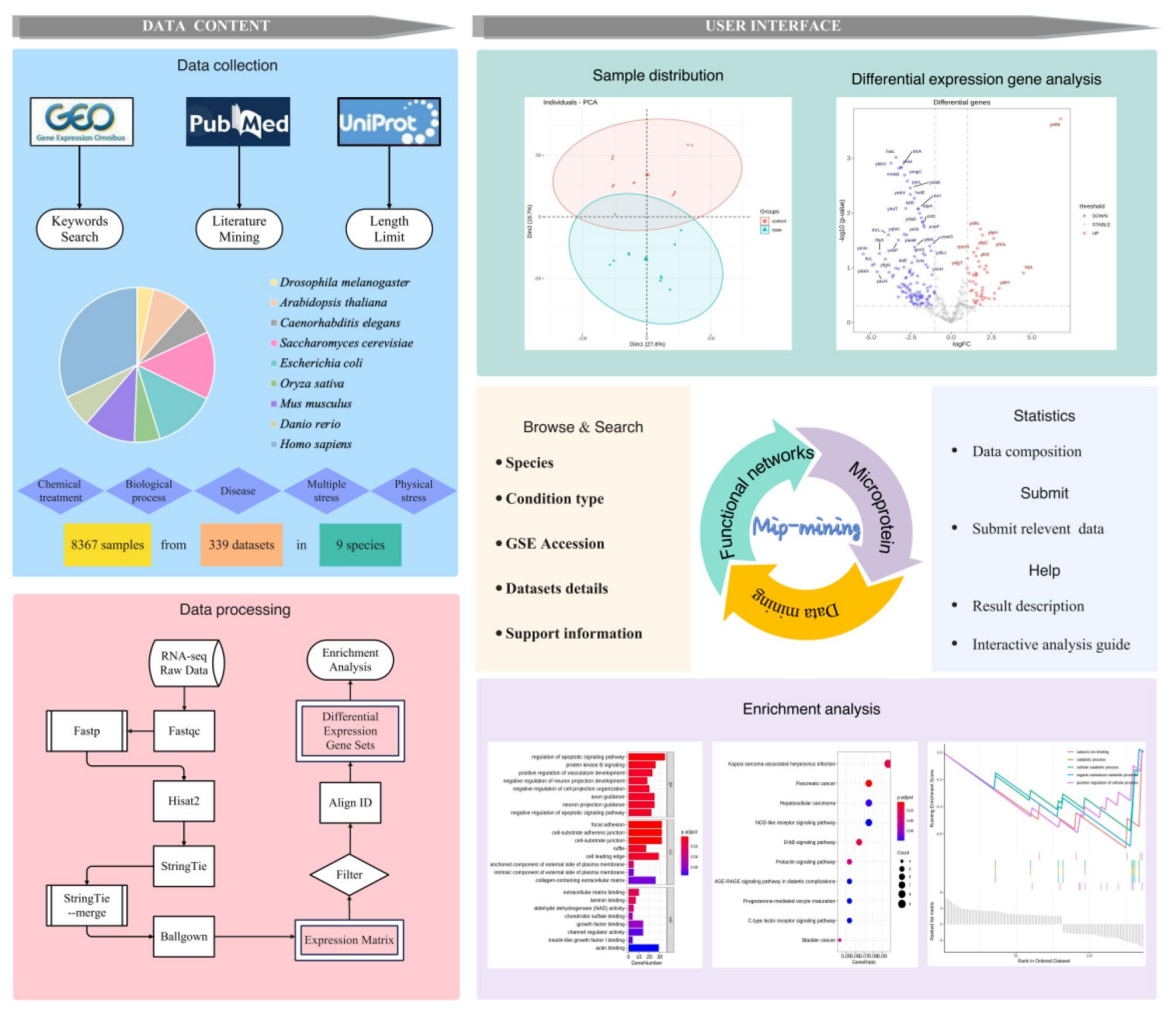
Schematic overview of the data acquisition and construction of the Mip-mining database. Items with blue and background represent data sources and data processing, respectively, whereas those with a light green background indicate sample distribution and differential expression gene analysis results. In addition, items with an off-white background correspond to the browse and search methods. The content of the statistics, submit, and help pages are shown in light blue, while the purple section represents enrichment analysis results

The schematic overview of the data acquisition and construction of the Mip-mining database is shown in Fig. 3. Firstly, data are collected from the GEO database with keyword searching; after a standardized RNA-seq analysis using Hisat2-stringTie-ballgown processing on HPC (High-Performance Computing) [55], the differential expression matrix is obtained. Then R packages are used for searching differentially expressed genes enrichment analysis and result visualization. All results can be downloaded locally.

**Fig. 4:**
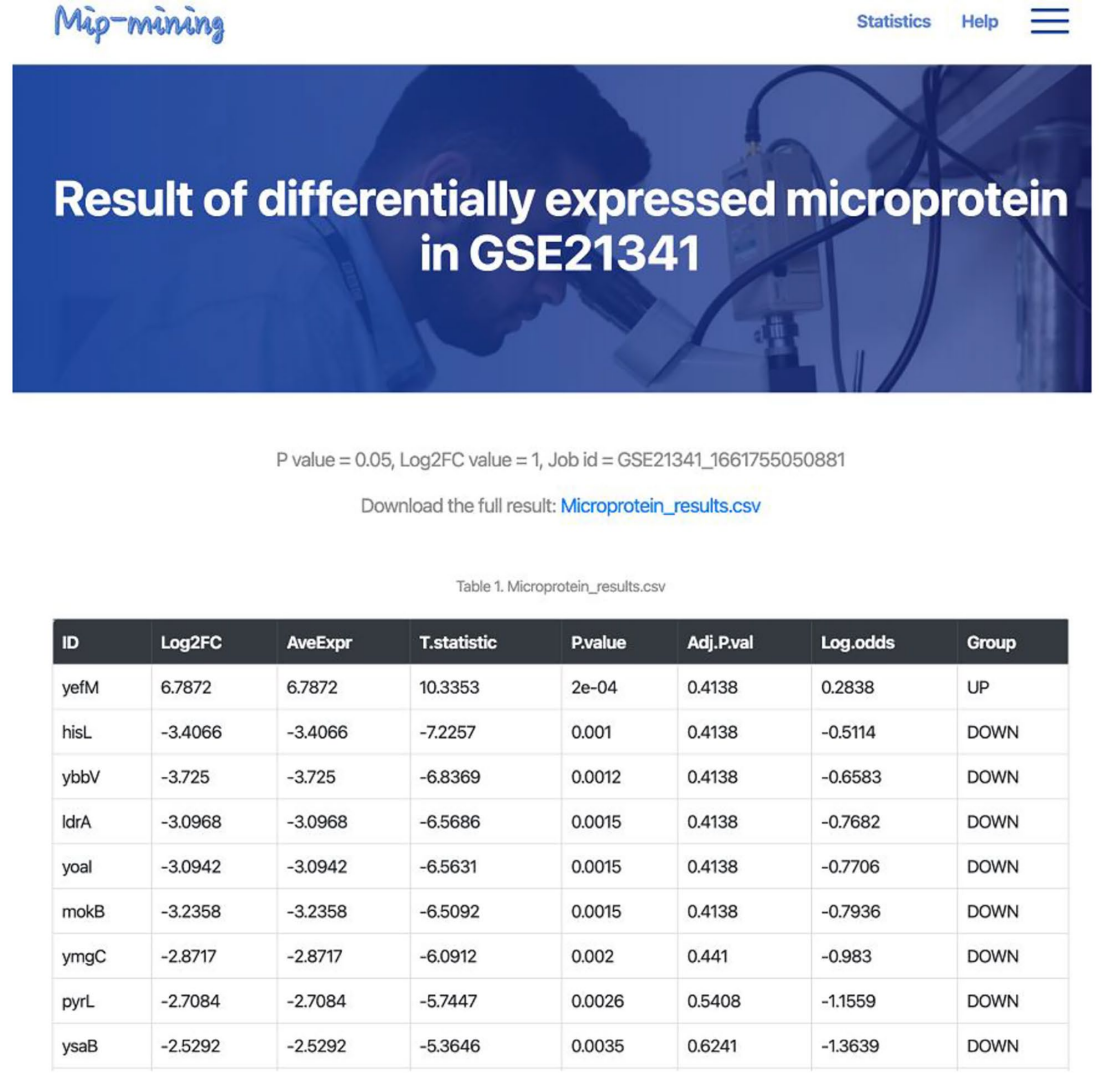
The first part of the Mip-mining database results page. DGEs (Differential Gene Expression) result table contains the microprotein gene names, Log2FC: an estimate of the log2-fold-change corresponding to the contrast (case vs. control), AveExpr: average log2-expression for the sample, T.statistic: moderated t-statistic, P.value: raw p-value, Adj.P.val: adjust corrected p-value, Log.odds: log-odds that the gene is differentially expressed and Group: gene label indicates up-regulation or down-regulation or stabilization of microprotein visualization of the sample distribution

**Fig. 5:**
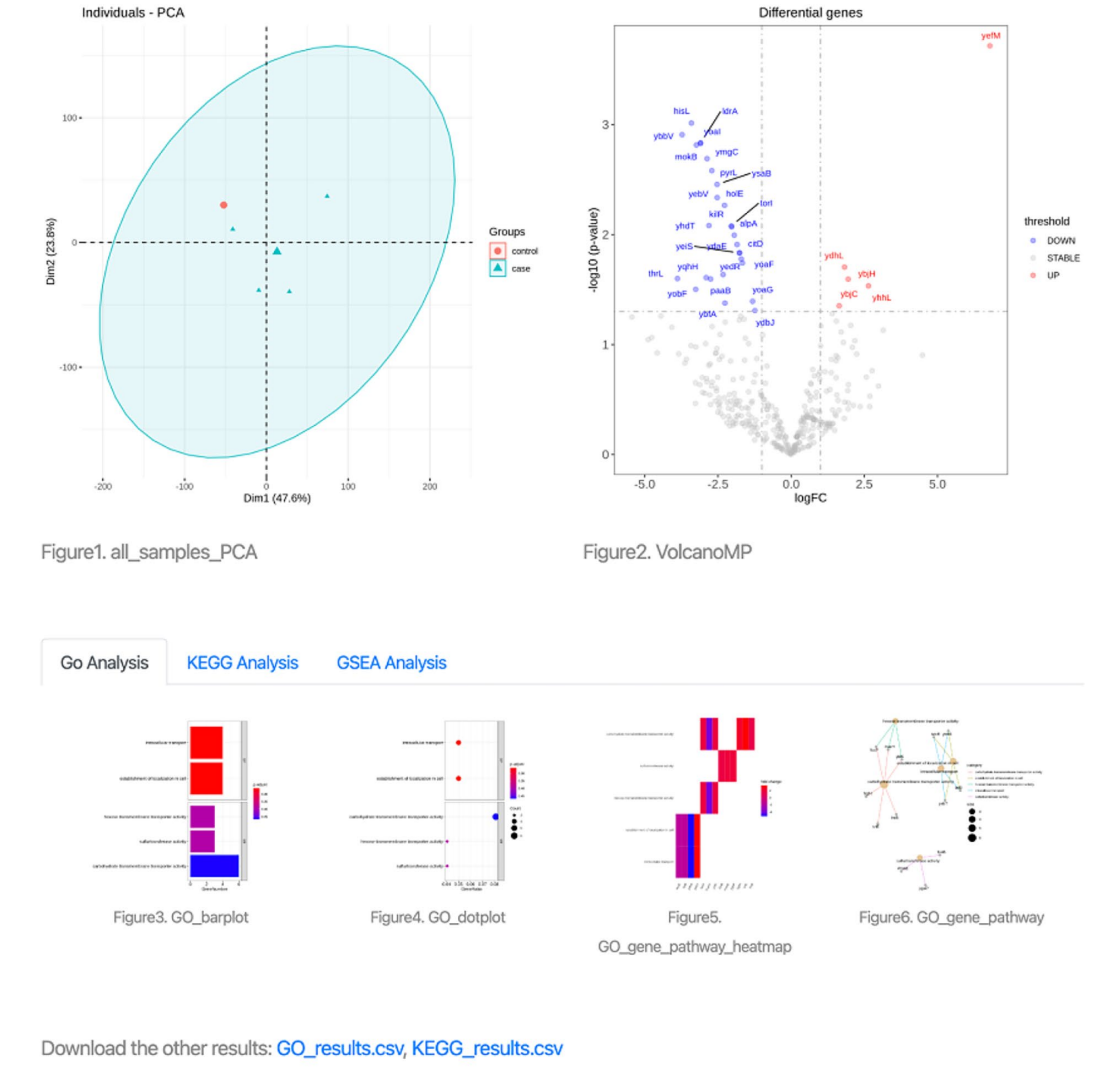
The second part of the result page. The page contains a visualization of sample distribution and DGEs (Differential Gene Expression) results, as well as the enrichment analysis results integrating GO, KEGG, and GSEA parts from RNA-seq data, and it also provides a download link for GO and KEGG analysis outcomes

The Mip-mining database benefits the establishment of the relationship between differential expression of microproteins and various conditions (including external environmental response and internal disease development). It would help to mine the corresponding functions of microproteins. Mip-mining provide three major functions: (i) Browse and search primary data through condition type, species, and GSE accession (Fig. 1. and Fig. 2.); (ii) Identification of differentially expressed microproteins and corresponding functional enrichment analysis (Fig. 4. and Fig. 5.); (iii) Result visualization, and download.

We next demonstrate the utility of Mip-mining in studies of microprotein functions in several species through the case studies below.

### Case studies

#### Case study 1. Stress tolerance-related microproteins in budding yeast *S. cerevisiae*

Yeast is commonly used in industries for food production, pharmaceutical research, chemical fermentation, and renewable energy production [56]. During bioproduction, yeast cells are subject to various stress conditions. For example, biorefinery of lignocellulosic biomass using yeast is negatively affected by decreased growth and metabolism due to inhibitors in biomass hydrolysate [57]. Among the inhibitors, acetic acid is commonly present and is highly toxic to yeast cells [58]. Improvement of acetic acid tolerance is thus desirable to develop yeast strains for efficient lignocellulosic biorefinery. In this regard, we used Mip-mining to analyze the expression of small proteins under acetic acid stress. We found that among the small proteins ranked in the GSE52160 data set analysis, three genes encoding microproteins showed significant changes (Table 2). As recorded in the *Saccharomyces* Genome Database (SGD) [59], the knockout of the microprotein gene *PMP2* directly affected growth under acetic acid stress, which supports that our database is functional in revealing microproteins with known roles. Furthermore, deleting *ATP15* and *SDH6* were reported to affect the growth of *S. cerevisiae* under low pH conditions and respiratory growth [60–62], respectively. The changed transcription level by the Mip-mining analysis indicates that *ATP*15 may be involved in acetic acid stress. To further examine whether *ATP15* is involved in acetic acid stress response, we overexpressed this gene using a high copy number plasmid pJFE3.

**Table 2:** Yeast microproteins identified by Mip-mining by analyzing the dataset of GSE52160^*^

Gene name	Log_2_FC		Phenotype and function
*PMP2*	1.94	Acetic acid resistance decreased by gene deletion	
*ATP15*	-2.92	Propionic acid pH resistance decreased by gene deletion	
*SDH6*	-3.4	Respiratory growth is absent after adding 2% acetate by deletion	

^*^Functions were retrieved from *Saccharomyces* Genome Database (SGD), https://www.yeastgenome.org/

#### The construction of ATP15 expressional plasmids and strains

The *ATP15* expression plasmid pJFE3 was constructed by introducing the *ATP15* gene cloned from *Saccharomyces cerevisiae* S288C into the high copy plasmid pJFE3 [63] between the sites of *TEF1p* and *PGK1t*. Then, the expression plasmid and the empty plasmid pJFE3 were transformed into the auxotroph *S. cerevisiae* BY4741 to produce the *ATP15* expressional strain BY4741-pJFE3-ATP15. The correctness of the expression plasmid and recombinant strain construction was verified by sequencing after PCR amplification. The primers used in PCR for construction and verification are listed in Table S1.

#### Allocation of medium

SC-Ura fluid nutrient medium: YNB(Yeast nitrogen base without amino acid with (NH_4_)_2_SO_4_)6.7 g/L, amino acid mixture without URA 0.77 g/L, and glucose 20 g/L. The prepared medium was sterilized by autoclave at 115℃ for 15 min. Acid stress medium is based on SC-Ura fluid nutrient medium, adding 1 M HCL to adjust the pH to 2.3. Acetic acid stress medium is prepared based on SC-Ura fluid nutrient medium, with the addition of 4.2 g/L acetic acid.

#### Strain inoculation and culture

The constructed overexpressed strain and control strain were reactivated by SC-Ura liquid medium two times, which were then added to SC-Ura fluid nutrient medium to obtain seed liquid. The activated strains were inoculated into shake bottles with the initial OD600 of 0.03, cultured at 30 °C shaking at 150 rpm. The broth was sampled at an appropriate time point to detect the growth under stress-free and stress conditions.

**Fig. 6:**
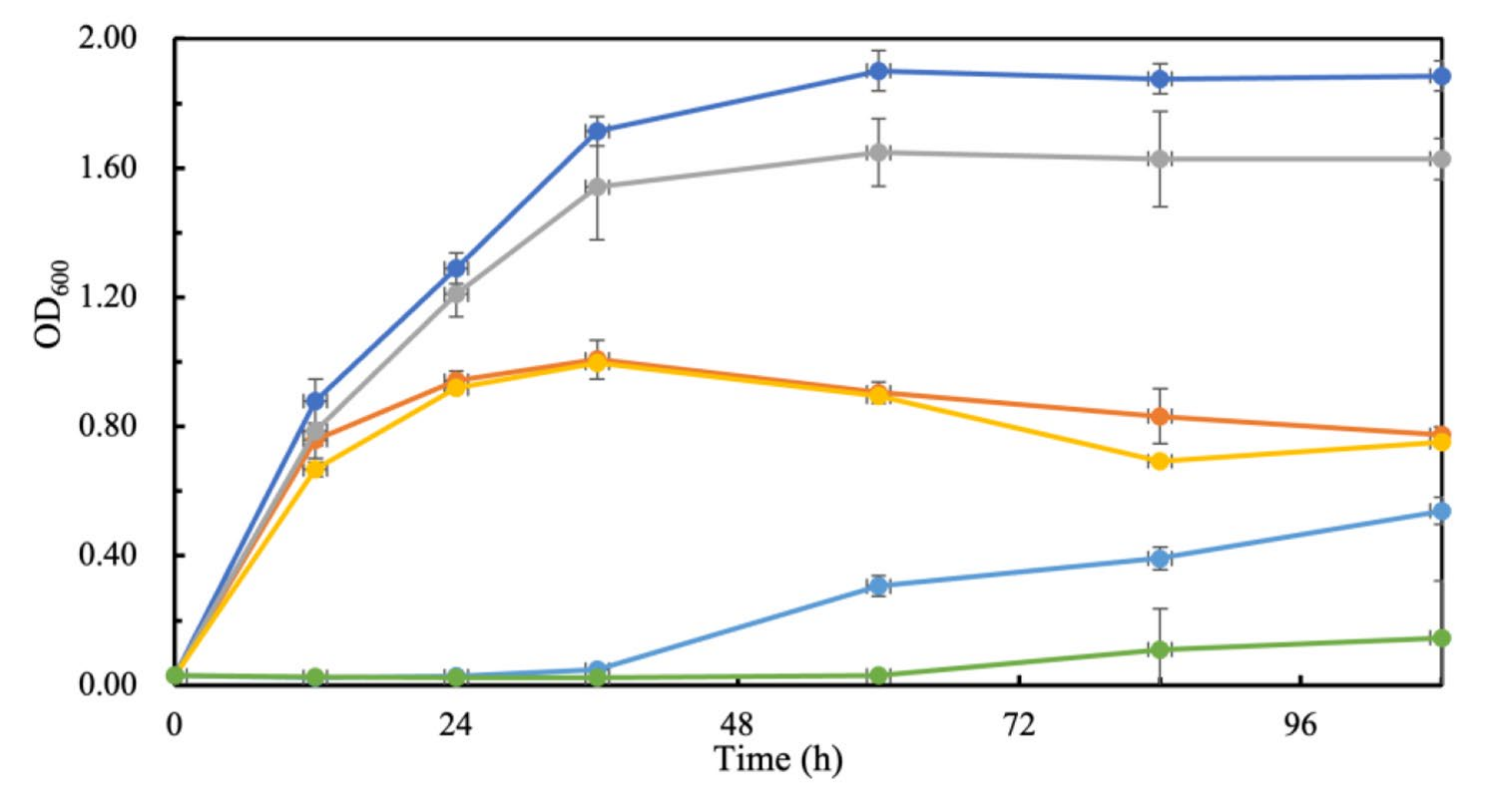
Overexpression of *ATP15* affects yeast growth and stress tolerance. Dark blue and dark orange, growth of the control strain and the *ATP15* overexpression strain carrying the empty plasmid and the *ATP15* expression plasmid, respectively, under stress-free conditions; Grey and light orange, growth of the control strain and the *ATP15* overexpression strain at pH 2.3; Light blue and green, growth of the control strain and the *ATP15* overexpression strain in the presence of 4.2 g/L acetic acid. Yeast strains were grown in YPD broth at 30 shaking at 150 rpm with or without addition of acetic acid, and pH 2.3 was adjusted using 1 M HCl.

The results revealed that high-level expression of *ATP15* severely inhibits growth in the presence of acetic acid; about 24 h longer lag phase time was observed when *ATP15* was overexpressed. Reduced biomass was observed under non-stress and low pH (2.3) conditions. The unprecedented growth repression by *ATP15* overexpression under acetic acid stress confirmed that this protein is critical in combating stress (Fig. 6.).

#### Case study 2. Microproteins in the model plant *A. thaliana*

Plant stress responses have been studied to provide a basis for breeding crops that resist salt, cold environment, drought, or microbial pathogens [18]. The temperature is an essential factor among these stress conditions encountered by plants. Low or high temperatures affect the development of plants and their immunity to harsh conditions [64]. In this regard, we select GSE116004 for analysis, which compares the global transcription of the model plant *A. thaliana* at 37 °C with the control condition at normal temperature (Table S1). We observed changes in *PIP1* and *PIP2*, which were annotated as endogenous secreted peptides that elicit an immune response and positive regulators of defence response [65]. So far, no reports have been found on the functions of these two proteins in heat resistance. Therefore, our results revealed the plant microproteins’ potential that can be further investigated for their functions under specific environmental conditions.

#### Case study 3. Microproteins related to human cancer

Breast cancer is a severe threat to women’s health, and triple-negative breast cancer is challenging to treat due to its lack of therapeutic targets, high recurrence rate, and uncomplicated metastasis. We selected the dataset GSE171957 to study the connection between microproteins and triple-negative breast cancer, hoping to provide more therapeutic directions for triple-negative breast cancer from the perspective of microproteins (Table S2). According to the results of the Mip-mining analysis, we conducted a literature survey and found that PKIB is involved in the signaling pathway induced by cAMP [66]. CENPW is associated with nucleosomes [67]. COA4 [68] is associated with cytochrome c oxidase. Among significantly down-regulated genes, long non-coding RNA SNHG12 has been proven to be a potential pan-cancer marker and therapeutic target [69]. NUPR1 promotes cancer cell metastasis, can help cancer cells adapt to the microenvironment after chemotherapy and play a role in drug resistance [70]. In addition, reducing RPS27L can regulate autophagy and promote tumorigenesis [71].In addition to microproteins directly associated with triple-negative breast cancer, we also found that significant downregulation of DPY30, which is thought to regulate the epithelial-mesenchymal transition to affect cervical squamous cell carcinoma [72], and is so far an unexplored microprotein regulator.

To summarize, through case studies of triple-negative breast cancer, we can find relevant key regulators that have been proven and can also provide researchers with more potential therapeutic targets and research directions.

## Discussion

Mip-mining in the current study is the first database focusing on transcriptome profiles in microproteins related to environmental stress tolerance or diseases. It will be useful for researching and applying microproteins in sustainable bioproduction, biomarker discovery, and disease treatment. Compared with the existing microprotein databases, including SmProt, sORFs.org, and PsORF, among others [23–31] contributing to the widespread existence of microproteins in living organisms, Mip-mining is unique because it aims to reveal the effects of microproteins under a wide range of conditions. The database contains expanded data set from more diverse organisms, which includes microorganisms, plants, and animals. Additionally, the data we collected focus on multiple stress conditions and various diseases, which enables the exploration of microproteins with essential functions. Besides, only high-quality transcriptomic data were collected, and most of the RNA-seq data have literature support for easy traceability, which guarantees the reliability of the analysis. Although most other databases collect data based on mass spectrometry analysis and ribosome profiling for microprotein studies, we emphasize that the transcription of microprotein genes contains essential information and cannot be neglected. Firstly, transcription regulation starts gene expression, and the co-transcription of microproteins and other genes correlates with their functions. Secondly, so far, detection of the translation of microproteins is still restricted by technical limitations due to low expression and or specific properties of microproteins; therefore, transcriptome data are a critical complement for in-depth studies.

The Mip-mining database establishes the connection between environmental stress or disease, microproteins, and functional characterization. Through analysis, it is possible to quickly clarify the changes in the mRNA level in the specific organism under each stress/disease condition, supported by multiple data sets. Enrichment analysis can help users to deduce which pathways are more important under certain conditions, and the data can be used to trace back which pathways small proteins are involved in. Compared with other related databases, our current database is more beneficial for researchers to establish functional exploration and design experiments for further mechanism studies.

The role of microproteins as regulatory proteins in various living organisms is increasingly recognized [73, 74]. However, studies on microproteins should not ignore the synergistic effects of these essential proteins with other proteins, such as the differential expression of multiple proteins simultaneously. Mip-mining provides a novel platform to explore protein interaction networks under various stressful environments involving microproteins. The information provided by our database can be further used to study protein interaction networks to design more powerful small proteins. In this regard, the results may help employ microproteins to assist large protein complexes in various life activities.

We provide the function of screening differentially expressed microproteins for each set of data, but the information supplement for each microprotein has not yet been completed. Links with other reference microprotein databases can supplement more microprotein-related information. Up to now, Mip-mining contains information about microproteins related to stress conditions in 9 species. With the emergence of more RNA-seq data from non-model organisms and the improvement and advancement of sequencing technology, we will continue to collect microprotein information of more other species and refine related external conditions, for example, more data related to various other human diseases.

## Conclusion

We present the Mip-mining database - an innovative tool that allows users to conduct personalized analysis of microprotein functions. The Mip-mining database hosts 336 sets of high-quality transcriptome data from 8626 samples and nine representative living organisms, including microorganisms, plants, animals, and humans. Microproteins are potentially related to various diseases and environmental stress conditions, including chemical, physical, biological, and multiple stresses, and thus understanding a related microprotein or set of microproteins is crucial for a thorough understanding of these conditions. Users can select specific cutoff values for enhanced customization of their analysis. Consequently, this tool serves as a valuable resource for research communities investigating microproteins in diverse scientific fields.

### Electronic supplementary material

Below is the link to the electronic supplementary material.

Supplementary Material 1

## Data Availability

All data, including preprocessed transcriptome data and filtered microprotein information are stored on the Mip-mining website (https://weilab.sjtu.edu.cn/mipmining/).

## Code Availability

Code for Mip-mining filtering is available at https://github.com/GlancerZ/Mipmining.
