# Supplementary material for: Exploring microproteins from various model organisms using the mip-mining database
Source: BMC Genomics. 2023 Nov 2;24:661. doi: 10.1186/s12864-023-09735-1 (PMC10623795; doi:10.1186/s12864-023-09735-1)
Supplement: Supplementary file 1 — Supplementary Material 1 [file 12864_2023_9735_MOESM1_ESM.docx]

*Supplemental information for*

**Exploring microproteins from various model organisms using the Mip-mining database**

Bowen Zhao^#a^, Jing Zhao^#a^, Muyao Wang^a^, Yangfan Guo^e^, [Aamir Mehmood](https://onlinelibrary.wiley.com/action/doSearch?ContribAuthorRaw=Mehmood%2C+Aamir)^a^, Weibin Wang^a^, Yi Xiong^a,f^, Shenggan Luo^a^, Dongqing Wei^*a,c,d^, Xinqing Zhao^*a^, Yanjing Wang^*a,b^

a. State Key Laboratory of Microbial Metabolism, Joint International Research Laboratory of Metabolic & Developmental Sciences, School of Life Sciences and Biotechnology, Shanghai Jiao Tong University, Shanghai, 200240, China.

b. Engineering Research Center of Cell & Therapeutic Antibody, School of Pharmacy, Shanghai Jiao Tong University, Shanghai, 200240, China.

c. Zhongjing Research and Industrialization Institute of Chinese Medicine, Zhongguancun Scientific Park, Meixi, Nayang, Henan, 473006, China.

d. Peng Cheng Laboratory, Vanke Cloud City Phase I Building 8, Xili Street, Nanshan District, Shenzhen, Guangdong, 518055, China.

e. Central Laboratory of Yan'an Hospital Affiliated to Kunming Medical University, Kunming, 650051, China.

f. Shanghai Artificial Intelligence Laboratory, Shanghai, 200232, China.

^*^Corresponding authors. Dong-Qing Wei, Xin-Qing Zhao and Yanjing Wang; Email addresses: [dqwei@sjtu.edu.cn](mailto:dqwei@sjtu.edu.cn), [xqzhao@sjtu.edu.cn](mailto:xqzhao@sjtu.edu.cn), [wangyanjing@sjtu.edu.cn](mailto:wangyanjing@sjtu.edu.cn)

^#^ Bowen Zhao and Jing Zhao contribute equally to this work.

**Data S1:** **Mipmining** **server tutorials**

**Table S1. Plant microproteins identified by Mip-mining**

**Table S2. Human microproteins related to cancer identified by Mip-mining**

**Data S1:** **Mipmining** **server tutorials**

1. **How to browse MiP-mining database?**

In the web page for specific species, high-throughput transcriptome data of microproteins are listed with basic information including the GSE Accession of the RNA-seq data in the GEO database (column “GSE Accession”), the species’ name of data (column “Species”), the condition type of the experiment (column “Condition type”), the sample number of the data (column “Sample numbers”) and the data source of the RNA-seq data including GSE title with the corresponding link (column “GSE title”).


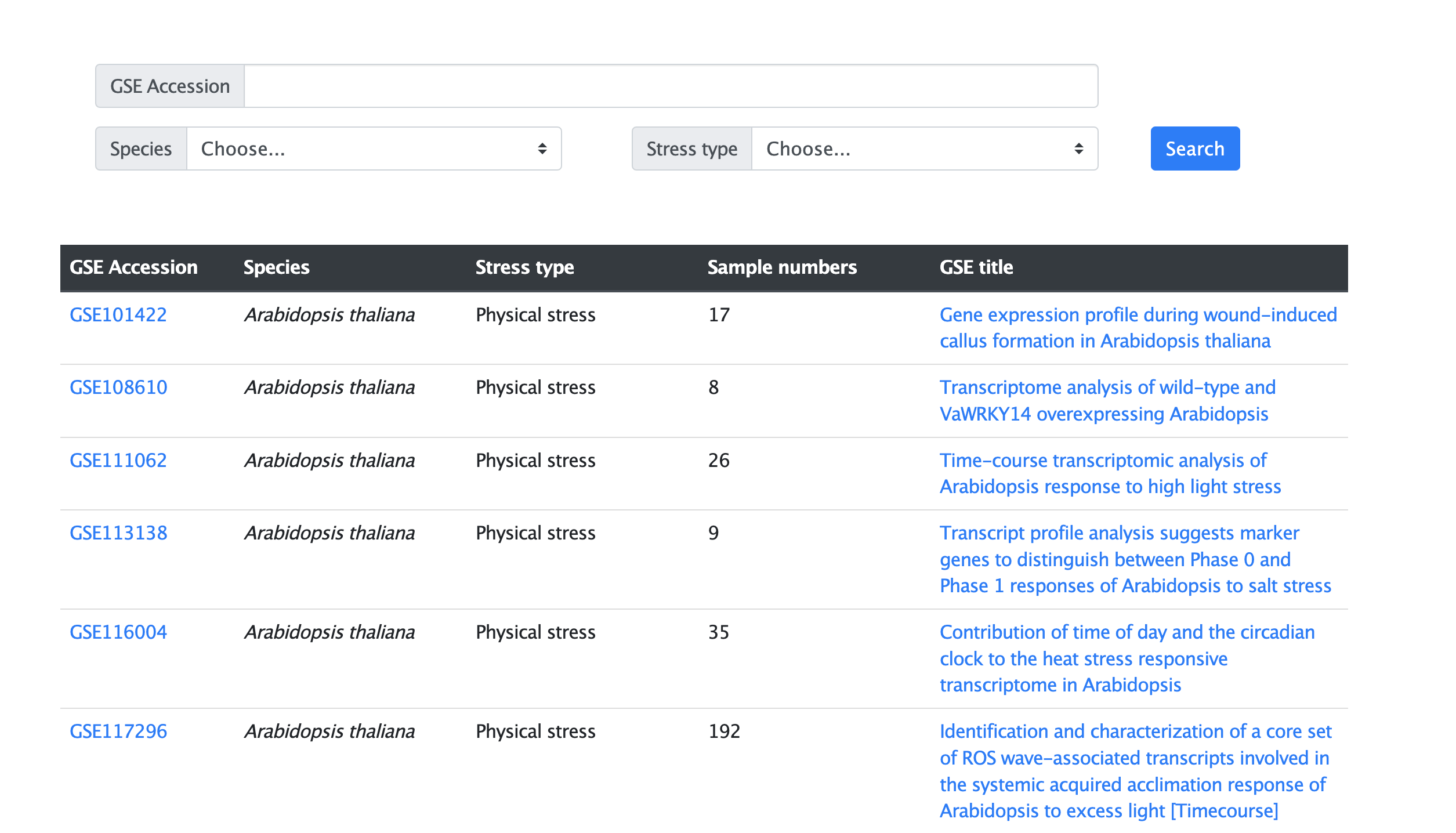


If users want to view the specific information of the RNA-seq data, they can click the corresponding GSE Accession Number to direct to the detailed information page. On the new page, detailed information about the specific RNA-seq data would be shown.


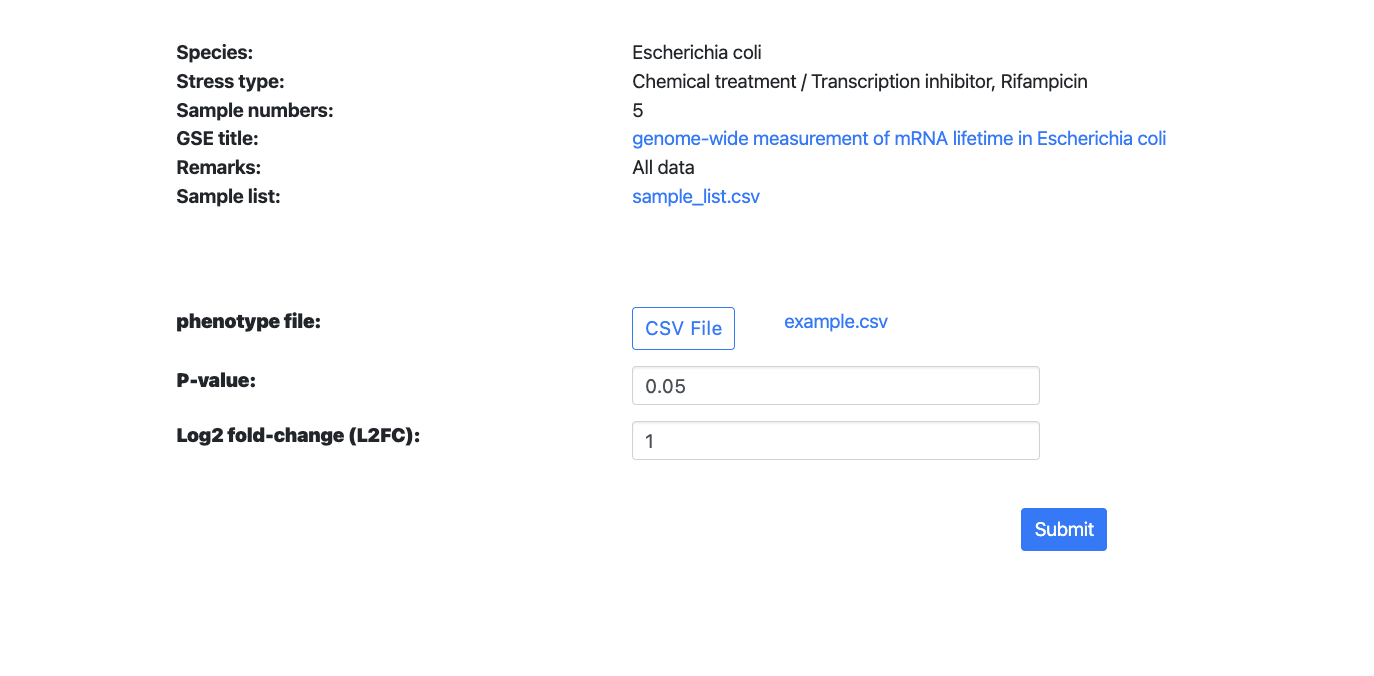


1. **How to search in Mip-mining database?**

The search page allows users to search species, condition type, and the corresponding GSE Accession Number stored in the database.

The search function includes three types ways, users can choose one of them to search:

1. Species search:

Users can choose species’ name, for example, “*Arabidopsis thaliana*” or other species collected in the database to inquire the RNA-seq data.


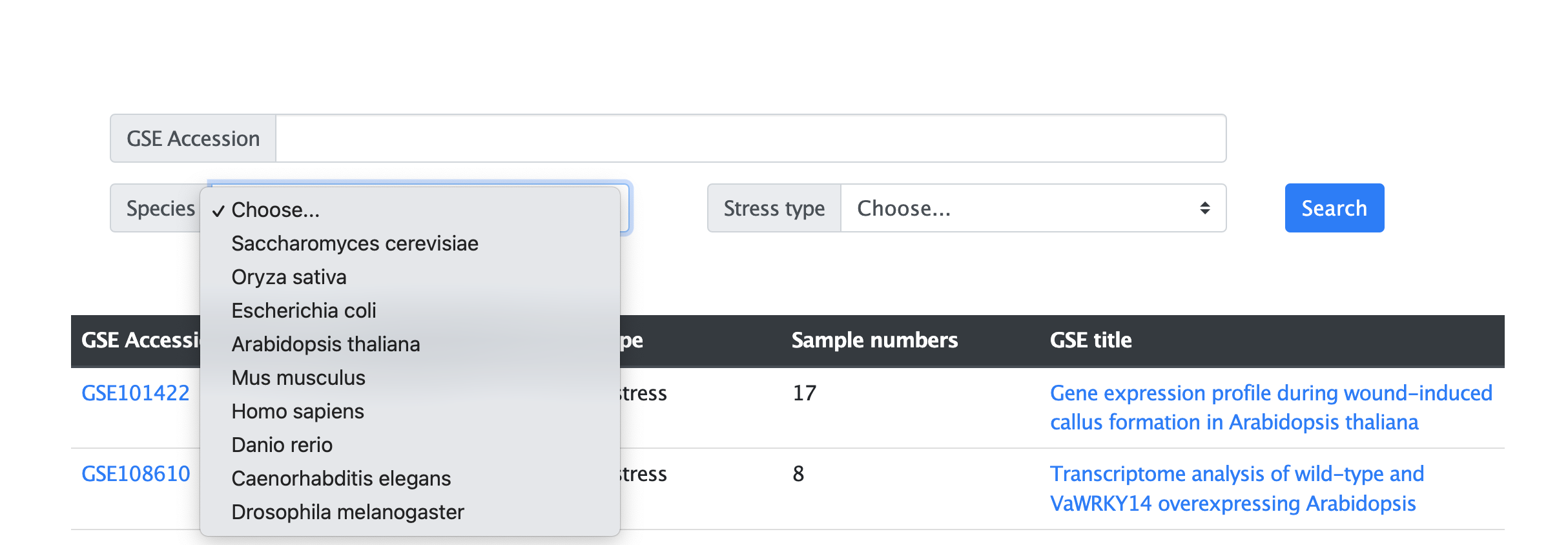


1. Condition type search:

Users can choose the conditions they are interested in to search, such as Biological response, Physical stress, Chemical treatment, or other condition.


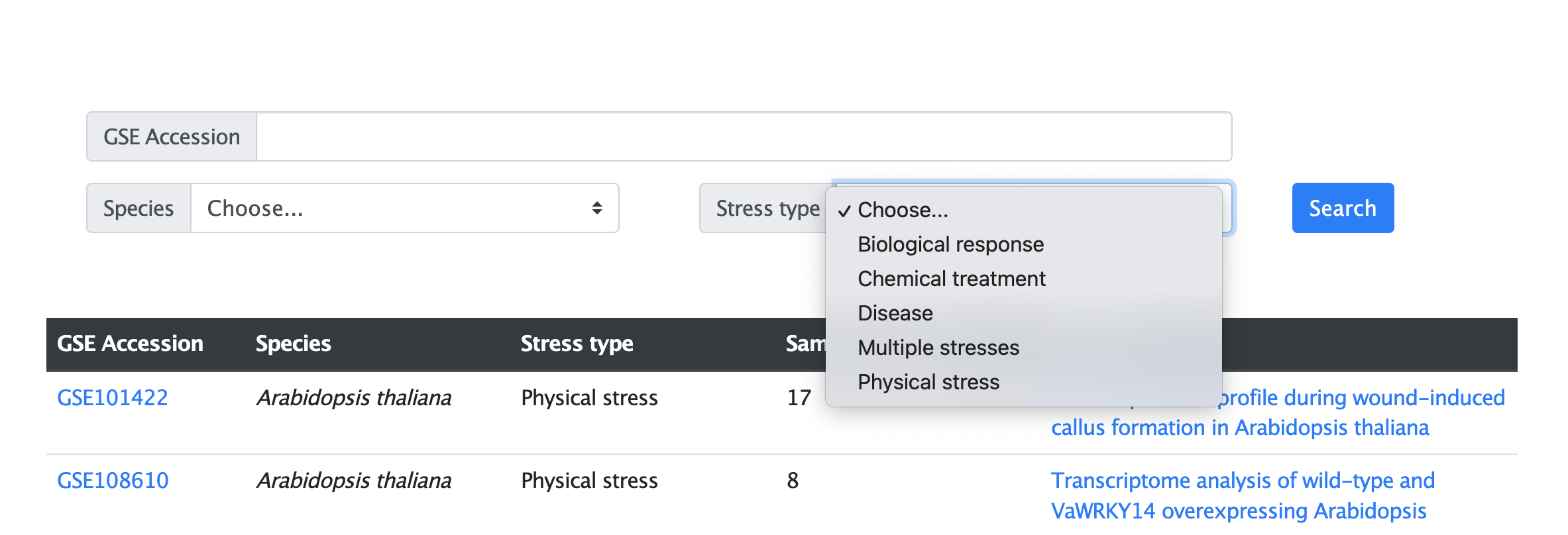


1. GSE Accession search:

Users can specify GSE Accession number for their search, such as GSE21341.

1. **How to analyze in Mip-mining database?**

The website provides an interactive analysis page. Users can perform online analysis by uploading the corresponding file and typing the threshold.


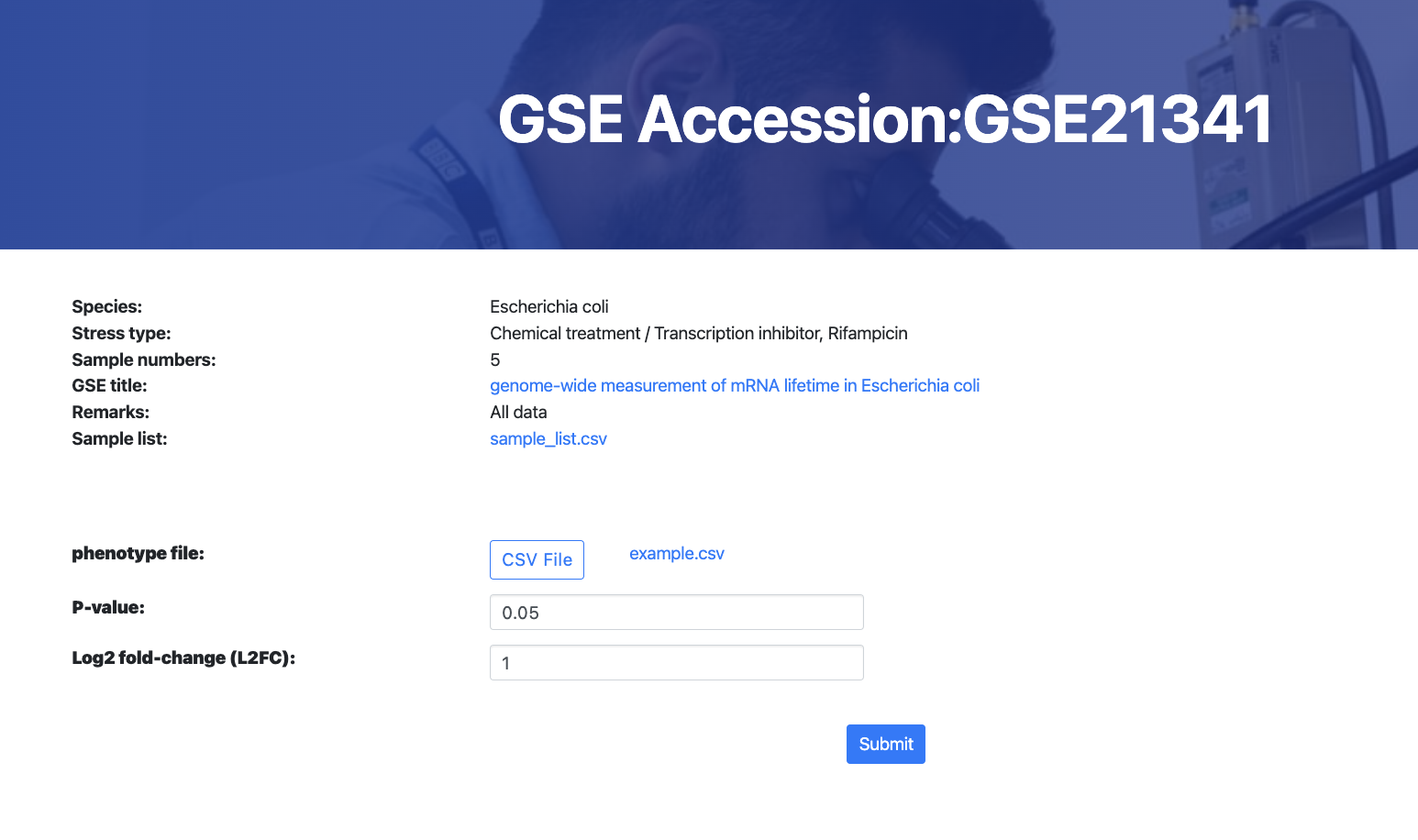


(1) Upload the phenotype file:

The user needs to determine the sample classification on the corresponding GEO page, and then upload the phenotype file in CSV format. Please ensure to upload the correct phenotype file, otherwise it will return error information. Users can download the reference phenotype file directly from the following github link: https://github.com/GlancerZ/Mipmining.

| id | Treatment |
| --- | --- |
| SRR057747 | control |
| SRR057748 | case |
| SRR057749 | case |
| SRR057750 | case |
| SRR057751 | case |

Example for the phenotype file of GSE21341

(2) Select threshold:

Users need to enter the p-value and log2 fold-change (log2FC) threshold to filter differentially expressed microproteins. Default parameters are log2FC 1.5, P-value 0.05.

(3) Submit：

When users finished the above steps, they can click the ‘submit’ button to start the personalized analysis.

**Table S1. Plant microproteins identified by Mip-mining**

| Gene name | Log2FC | Functions |
| --- | --- | --- |
| *SAUR15* | -1.92 | Functions as a positive effector of cell expansion through modulation of auxin transport |
| *PSK4* | 1.54 | Promotes plant cell differentiation, organogenesis and somatic embryogenesis as well as cell proliferation. |
| *PIP2* | -2.26 | Endogenous secreted peptide that acts as elicitor of immune response and positive regulator of defense response. Acts as negative regulator of root growth. |
| *PIP1* | 3.35 | Endogenous secreted peptide that acts as elicitor of immune response and positive regulator of defense response. Acts as negative regulator of root growth. |
| *PSK5* | -1.56 | Promotes plant cell differentiation, organogenesis and somatic embryogenesis as well as cell proliferation (By similarity).May be involved in the low quiescent center cell proliferation |

**Table S2. Human microproteins related to cancer identified by Mip-mining**

| Gene name | Log2FC | Functions |
| --- | --- | --- |
| *PKIB* | 1.09 | Involved in the signaling pathway induced by cAMP |
| *CENPW* | 1.10 | Associated with nucleosomes |
| *COA4* | 2.73 | Associated with cytochrome c oxidase |
| *SNHG12* | -1.48 | A potential pan-cancer marker and therapeutic target |
| *NUPR1* | -1.18 | Promotes cancer cell metastasis and can help cancer cells adapt to the microenvironment after chemotherapy, and play a role in drug resistance |
| *RPS27L* | -1.04 | Regulate autophagy and promote tumorigenesis |
| *DPY30* | -3.28 | Regulate epithelial-mesenchymal transition to affect cervical squamous cell carcinoma |
